# DOES EDUCATIONAL ATTAINMENT IMPROVE COGNITIVE FUNCTIONING OF OLDER TRIBAL POPULATION IN INDIA?

**DOI:** 10.1093/geroni/igae098.0194

**Published:** 2024-12-31

**Authors:** Neha Kumari, Nandita Saikia

**Affiliations:** International Institute for Population Sciences, Mumbai, Maharashtra, India; International Institute for Population Sciences, Mumbai, Maharashtra, India

## Abstract

Enhanced cognitive functioning is closely linked with the overall health and well-being of the elderly population. The pivotal role of education in determining cognitive capabilities is widely recognized. Particularly, tribal communities, who often endure vulnerability and marginalization due to limited educational access, predominantly reside in rural Indian regions, magnifying their susceptibility to cognitive decline. Our study delves into the influence of education on cognitive functioning among tribal older adults in India. Our investigation draws on secondary data from the nationally representative Longitudinal Ageing Study in India (2017-18), encompassing 62,322 adults aged 45+ years. We employed continuous assessments of five cognitive domains to gauge cognitive functioning, adapted from the Mini-Mental State Examination (MMSE). Linear regression analysis and decomposition analysis unveiled the major associates and contributors of cognitive disparities between tribal and non-tribal cohorts. Regression results revealed significantly lower odds of better cognitive functioning among individuals in the tribal category compared to non-tribal counterparts (Coefficient: -1.33; p < 0.001). Decomposition analysis illuminated that a considerable 77.5% (Coefficient: -2.73; p-value < 0.001) of the divergence attributed to caste-based differences was elucidated by varying characteristics. Addressing the educational chasm between tribal and non-tribal adults could potentially ameliorate cognitive inequity by 39%, while narrowing the rural-urban residence divide could contribute an additional 8% reduction. Notably, among others, the absence of education emerges as a pivotal factor in the cognitive underdevelopment within India’s tribal populace. Tailored educational policies targeted at tribal communities promise to foster cognitive growth and defer cognitive health deterioration.

